# Effects of Social Media Use on Brain Dynamics in Young Male Adults: Multistage Concurrent Electroencephalography–Functional Magnetic Resonance Imaging Study

**DOI:** 10.2196/86963

**Published:** 2026-08-06

**Authors:** Ke Bai, Bin-Jie Wu, Ze-Ze Wang, Xu-Jia Cao, Jia-Qi Dou, Xin-Yue Jiang, Sha-Sha Zhao, Zhu-Hong Chen, Yu-Ting Li, Yu-Xuan Shang, Guan Yang, Yu Han, Xiu-Long Feng, Bo Hu, Wen Wang, Tian-Xiang Zhou

**Affiliations:** 1 Department of Radiology, Tangdu Hospital, The Fourth Military Medical University, 569 Xinsi Road, Xi’an, Shaanxi, China, 86‑29‑84778689; 2 Functional and Molecular Imaging Key Lab of Shaanxi Province, Xi'an, Shaanxi, China; 3 7T MRI Precision Neurology Platform of Shaanxi Province, Xi'an, Shaanxi, China; 4 Institute of Basic Medicine, The Fourth Military Medical University, Xi'an, Shaanxi, China

**Keywords:** social media use, SMU, functional magnetic resonance imaging, fMRI, dynamic amplitude of low-frequency fluctuations, dALFF, dynamic functional connectivity, dFC, electroencephalography, EEG microstates

## Abstract

**Background:**

Noninvasive brain stimulation may alleviate social media addiction, but its efficacy requires accurate individual targeting and real-time brain monitoring. The neural mechanisms underlying the effects of social media use (SMU) remain unclear, limiting the development of interventions. Understanding how different levels of SMU modulate brain activity could guide personalized neuromodulation strategies.

**Objective:**

This study investigated a cohort of young adults using a multistage design, with concurrent electroencephalography (EEG) and functional magnetic resonance imaging (fMRI) to examine the effects of SMU on brain activity. It aimed to characterize static and dynamic neural changes at baseline and after a standardized SMU task in individuals with different daily SMU durations.

**Methods:**

Participants were all male and were divided into a heavy social media users (HSMU) group and a light social media users (LSMU) group based on self-reported daily SMU duration. All participants underwent baseline fMRI scanning, followed by an EEG-fMRI session immediately after a 2-hour controlled SMU task. Analyses were performed on the static and dynamic amplitudes of low-frequency fluctuations (sALFF and dALFF), static and dynamic functional connectivity (sFC and dFC), and EEG microstates.

**Results:**

At baseline, compared with the LSMU group, the HSMU group showed lower dALFF variability in the middle frontal gyrus. After the immediate-effect task, the LSMU group exhibited increased sALFF in the temporal lobe and decreased sALFF in the middle and superior frontal gyri. The HSMU group showed increased sALFF in the middle temporal gyrus and decreased sALFF in the inferior temporal gyrus, superior parietal gyrus, and prefrontal cortex. Regarding dALFF variability, the LSMU group showed a decrease in the superior medial frontal gyrus, whereas the HSMU group showed a decrease in the middle frontal gyrus and an increase in the calcarine cortex. sFC analysis revealed increased connectivity across nearly all networks in the LSMU group. Conversely, the HSMU group showed reduced sFC between the visual and default mode networks. The HSMU group showed significantly shorter duration of microstate A, shorter duration and lower coverage of microstate C, and longer duration and higher coverage of microstate D.

**Conclusions:**

This study is the first to characterize the distinct neural patterns associated with different levels of daily SMU, using both EEG and fMRI to assess sALFF and dALFF alterations, widespread functional connectivity changes, and EEG microstate reorganizations. These findings demonstrate the unique value of multimodal assessment in identifying potential neural targets for personalized neuromodulation in social media addiction. Future studies should explore whether modulating these identified neural markers can effectively alleviate addictive behaviors and improve clinical outcomes across diverse populations.

## Introduction

Excessive social media use (SMU) can induce psychiatric symptoms, including anxiety [1,2], depression [3,4], and insomnia [5], as well as cognitive disturbances such as impaired attention, cognitive control, and memory function [6-8]. Noninvasive neuromodulation techniques such as transcranial magnetic stimulation (TMS) and transcranial electrical stimulation (tES) show promise in treating social media addiction but require precise targeting of specific brain regions and the decoding of neural activity to observe treatment-responsive brain dynamics [9]. For instance, during TMS treatment for nicotine or alcohol addiction, functional magnetic resonance imaging (fMRI) monitoring of prefrontal-striatal circuitry responsiveness has been shown to enhance therapeutic efficacy [10,11]. Consequently, comprehensive investigations are needed to decode the neural signatures of SMU, thereby facilitating the development of targeted neuromodulation.

Previous fMRI studies have shown that SMU is associated with altered functional connectivity (FC) in the right inferior frontal gyrus [12], a key region implicated in cognitive control processes that appears particularly vulnerable in adolescents exhibiting problematic smartphone use. Additionally, converging evidence points to functional alterations in the brain associated with SMU in both the ventral attention network [13,14] and the default mode network (DMN) [13,14]. However, the predominance of correlational designs means that causal relationships between SMU and neurobiological changes cannot be established. Consequently, the current evidence base remains insufficient to guide the development of precisely targeted neuromodulation.

Critically, most existing studies rely on temporally averaged fMRI signals, which inherently lack the millisecond-scale temporal resolution required for real-time optimization of TMS or tES protocols [15]. While dynamic fMRI analysis has emerged as a valuable tool for characterizing variability and stability in brain activity [16], the intrinsic hemodynamic response delay fundamentally limits its ability to capture rapid dynamics in neural activity. In contrast, electroencephalography (EEG) microstate analysis provides millisecond-level temporal precision, offering unique insights into the temporal organization of resting-state networks [17,18]. Based on our previous work on EEG microstates [19,20] and evidence demonstrating FC plasticity due to SMU [21], including abnormal connectivity among the DMN, visual network (VN), and bilateral frontoparietal network [22], integrating EEG temporal resolution with fMRI spatial resolution may help further reveal brain alterations associated with SMU.

To address these critical gaps in temporal resolution and characterization of neural responses, the present study employed a multistage experimental design to investigate the effects of SMU on neural activity. Unlike conventional studies that employ static or dynamic metrics and EEG microstates in isolation, we integrated these techniques within a synergistic multimodal framework to capture brain function across temporal scales. Specifically, we first used fMRI to examine differences in neural substrates associated with varying daily SMU durations. We then used simultaneous EEG-fMRI acquisition to capture the immediate neurobiological effects following a 2-hour controlled SMU exposure. This study contributes to the decoding of neural activity and network alterations associated with SMU at both second- and millisecond-level resolutions.

## Methods

### Participant Recruitment and Grouping

A total of 175 participants from Fourth Military Medical University were recruited and completed a self-report questionnaire covering demographic information (including age, sex, height, weight, smoking habits, drinking habits, color blindness, handedness, history of traumatic brain injury, and family history of mental illness) and daily SMU duration. SMU was defined as the time spent on the 10 most widely used social media platforms in China [23]. Participants were excluded if they had a BMI greater than 30 or less than 18.5; smoked or consumed alcohol; had color blindness; were left-handed; had a history of traumatic brain injury; or had a family history of mental illness. Due to a significant sex imbalance in the initial cohort (only 8 out of 175 participants were female participants) and the fact that only 3 female participants agreed to participate in the multistage research, only male participants were included in the final sample to prevent potential sex-related bias. The final analysis included 30 heavy social media users (HSMU) and 19 light social media users (LSMU), based on the distribution of daily SMU duration among the participants. Detailed grouping information is provided in Multimedia Appendix 1.

### Study Design

To prevent bias arising from the use of different applications, browsing microblogs on a smartphone was defined as the social media task. The microblogging platform Weibo (Sina Corporation), which functions similarly to Facebook, is one of China’s most commonly used social media applications. The experimental design consisted of the following steps: (1) all participants underwent an initial fMRI scan at baseline status; (2) all participants completed a 2-hour short-term SMU task and underwent a simultaneous fMRI-EEG scan immediately thereafter. Given the complexity of long-term effects in neurobehavioral research, this study did not include long-term follow-up tasks.

### Data Acquisition

fMRI data were acquired using a Discovery MR750 (GE HealthCare) 3.0-T scanner with an 8-channel phased-array head coil. Data were obtained using gradient-recalled echo planar imaging sequences with the following parameters: repetition time (TR)=2000 ms, echo time (TE)=30 ms, flip angle=90°, slice number=36, gap=1 mm, field of view=220×220 mm^2^, matrix=64×64, slice thickness=3 mm, in-plane spatial resolution=3.44×3.44 mm^2^, and time points=185. Detailed scanning parameters for T1-weighted imaging are provided in Multimedia Appendix 1. Simultaneous EEG data were recorded using a 64-channel geodesic sensor net–HydroCel system (NetStation software; Electrical Geodesics, Inc) with an equidistant geodesic sensor net layout with MagLink (Electrical Geodesics, Inc) magnetic resonance (MR)–compatible amplifier. The MR-compatible amplifier was placed outside the scanner room, and the sampling rate was set at 500 Hz. Electrode impedances were reduced to below 20 kΩ before recording. The EEG recording was synchronized with the MR scanner’s internal clock. During all resting-state fMRI and simultaneous EEG acquisitions, participants were instructed to keep their eyes closed.

### fMRI Preprocessing

Imaging data preprocessing was conducted using Statistical Parametric Mapping (SPM12) [24] and the DPABI toolkit [25] running on MATLAB 2022b (MathWorks, Inc). The preprocessing steps included the following: (1) removing the first 10 time points to stabilize the scanning signal; (2) slice-timing correction; (3) head-motion correction, excluding images with head motion translation >2.0 mm or rotation >2°; (4) normalization to the Montreal Neurological Institute (MNI) space and resampling to 3×3×3 mm³ resolution; (5) spatial smoothing using an 8-mm full-width at half-maximum Gaussian kernel; (6) regression of nuisance covariates, including white matter and cerebrospinal fluid signals; (7) linear detrending to remove low-frequency drift; and (8) bandpass filtering performed between 0.01 and 0.08 Hz.

### fMRI Neural Activity Analysis

**Figure 1.**
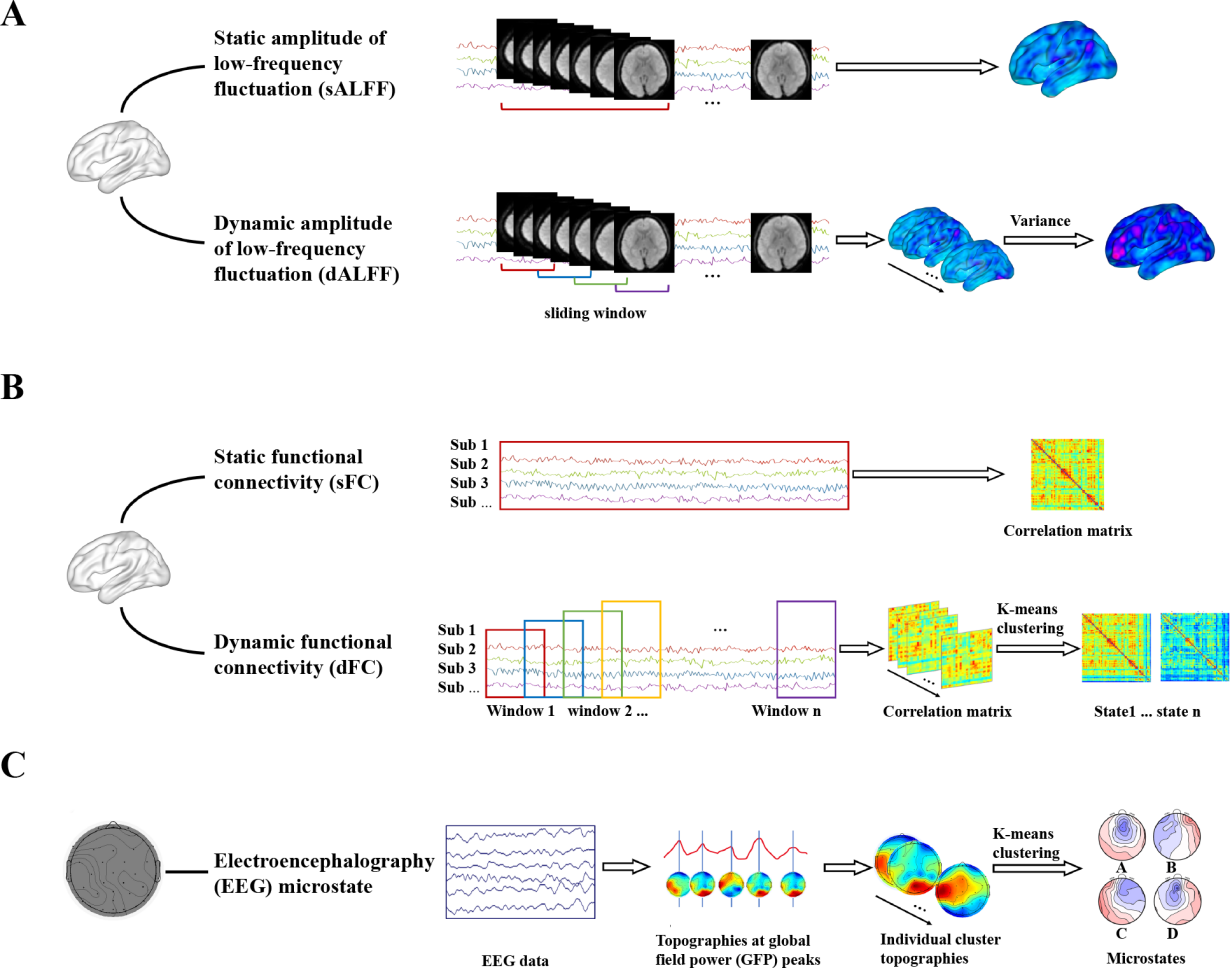
Data processing flow. (A) sALFF and dALFF analyses processing. Compared with sALFF, dALFF uses a sliding window method to obtain multiple windows. After setting the window size and stride length, the preprocessed time series were divided into multiple windows, and a series of amplitude of low-frequency fluctuation maps was acquired. The SD was subsequently used to calculate the variance of the dALFF. (B) sFC and dFC analyses processing. Compared with sFC, dFC uses the sliding window method to obtain multiple windows and has set a constant window size and stride length. In each window, Pearson correlation coefficients between regions of interests were calculated to obtain correlation matrices. (C) EEG microstate analysis processing. After the EEG data were preprocessed, the GFP was calculated for each participant. The k-means clustering was performed at the GFP peaks to generate a topographic map of the GFP peak. Finally, microstates were obtained.

Figure 1A shows the static amplitude of the low-frequency fluctuations (sALFF) and the dynamic amplitude of low-frequency fluctuations (dALFF) analysis processing flow. The sALFF was calculated using the DPABI toolkit and *z* score transformed to enhance normality. The dALFF was calculated using the sliding window method with the DynamicBC toolbox [26]. The selection of window length is a key consideration in dynamic analysis, as it governs the temporal resolution of the brain dynamics being measured and remains a topic of ongoing discussion. Previous studies have adopted a wide range of window lengths, from as short as 10 s [27] to as long as 180 s [28]. Within this empirically validated range, a window size of 40 TRs was selected in the current study with a sliding stride length of 1 TR [29,30]. Auxiliary analyses with window sizes of 30 TRs, 50 TRs, 60 TRs, and 70 TRs were also performed (results are provided in Multimedia Appendix 1). To evaluate the temporal variability of brain activity, we calculated the SD of the dALFF maps. Finally, the dALFF variability was *z* score transformed. The dALFF maps were subsequently spatially smoothed using a 6-mm full-width at half-maximum Gaussian kernel. Figure 1B illustrates the pipeline for static functional connectivity (sFC) and dynamic functional connectivity (dFC) analyses. For each participant, the average time series for brain regions was extracted using the Automated Anatomical Labeling atlas, and FC between regions was computed using Pearson correlation. dFC was computed using the DynamicBC toolbox (with parameters matching the dALFF analysis) and then clustered using k-means with the L1 distance function. The number of clusters was determined by the elbow criterion, with centroids representing dFC states. For each dFC state, 3 temporal characteristics were computed, including the fractional rate (the percentage of windows in each state), the mean dwell time (the average number of consecutive windows sustained in each state), and the state-transition probability (the probability of switching between states).

### EEG Preprocessing

Offline preprocessing was performed using EEGLAB (electroencephalography laboratory) and the FMRIB (functional magnetic resonance imaging of the brain) plug-in from Daniel Arzate-Mena et al [31]: (1) gradient artifacts were removed using FASTR (functional magnetic resonance imaging artifact slice template removal) [32]; (2) QRS complexes were detected from the electrocardiogram channel [33]; (3) ballistocardiographic artifacts were removed using the optimal basis set method; (4) channel locations were imported, unused electrodes were removed, and continuous EEG data were re-referenced to the common average reference; (5) data were then band-pass filtered at 0.1‐45 Hz, and channels with excessive noise were interpolated; and (6) independent component analysis was performed to identify and remove components related to eye movements, muscle activity, and other artifactual sources, with component rejection confirmed by visual inspection [19,34].

### EEG Microstate Analysis

Figure 1C illustrates the microstate analysis pipeline. The cleaned EEG data were further band-pass filtered between 2Hz and 20 Hz and processed using the Microstate Analysis Toolbox for EEGLAB. For each participant, global field power was computed from the preprocessed EEG signals. Topographic maps were extracted at global field power peak time points [35], followed by k-means clustering [36]. A cluster number range of 2 to 11 was tested, with microstate polarity disregarded. Based on cross-validation criteria established in prior studies [35,37], 4 microstate maps were selected as optimal. Four topographic map classes labeled A, B, C, and D were identified for each participant, and grand mean templates were generated across all participants. Spatial correlations were calculated between each participant’s topographic maps and the templates, with each map assigned to the microstate class showing the maximum absolute Pearson correlation. For each microstate class, three key parameters were quantified: (1) duration (mean temporal span in milliseconds), (2) occurrence (frequency per second), and (3) coverage (temporal predominance percentage).

### Statistical Analysis

SPSS 26.0 (IBM Corp) was used for statistical analysis. Demographic comparisons between the 2 groups were conducted using the 2-sample *t* test with 2‑tailed significance. For neuroimaging measures, parametric tests were used when the data were normally distributed, whereas nonparametric tests were applied when they were not. In the case of dALFF and sALFF, between-group comparisons (HSMU vs LSMU) were performed using independent-samples *t* tests, and within-group pretask vs posttask comparisons were assessed using paired *t* tests. Gaussian random field (GRF) theory was used for multiple comparison correction, with a voxel-level threshold of *P*<.005 and a cluster-level threshold of *P*<.05. The sFC was compared using a paired *t* test (2-tailed) combined with the network-based statistics (NBS), with a permutation test (5000 times) for multiple comparison correction (*P*<.05). The Mann-Whitney *U* test was used to compare differences in EEG microstate parameters between the HSMU and LSMU groups, and because these analyses were exploratory, they were not corrected for multiple comparisons.

### Ethical Considerations

This study was approved by the Institutional Review Board of the Fourth Military Medical University (approval number GKJ-Y-202303‐131). All procedures involving human participants were performed in strict accordance with the Declaration of Helsinki. Written informed consent was obtained from all participants before enrollment. To protect participant privacy, all data were deidentified before analysis, and no personally identifiable information was retained. Participants did not receive any financial or nonfinancial compensation for their participation in this study. No identifiable personal information or personal user images are included in the manuscript or supplementary materials.

## Results

### Demographic Information

Table 1 shows the demographics, and there was no significant difference between the 2 groups except for time spent on social media.

**Table 1.** Demographic characteristics and social media usage.

Characteristics	HSMU^a^	LSMU^b^	*P* value
Demographics			
Age (y), mean (SD)	21.13 (0.72)	20.79 (1.01)	.21
Sex, n			
Male	30	19	—^c^
Female	0	0	—
Height (cm), mean (SD)	175.10 (4.25)	174.21 (6.67)	.61
Weight (kg), mean (SD)	67.15 (6.31)	67.45 (7.66)	.88
BMI (kg/m^2^), mean (SD)	21.89 (1.81)	22.18 (1.63)	.57
Smartphone usage (h), mean (SD)			
Social media	2.05 (0.85)	0.41 (0.36)	<.001^d^
Film and television	0.29 (0.59)	0.21 (0.59)	.65
Chatting software	0.98 (0.91)	0.87 (0.89)	.68
Online shopping	0.10 (0.23)	0.06 (0.22)	.55
Online game	0.55 (0.52)	0.53 (0.42)	.88
E-book	0.33 (1.11)	0.39 (0.74)	.73

a HSMU: heavy social media users.

b LSMU: light social media users.

c Not applicable.

d *P*<.001.

### Baseline Differences in dALFF Variability Between the HSMU and LSMU Groups

**Figure 2.**
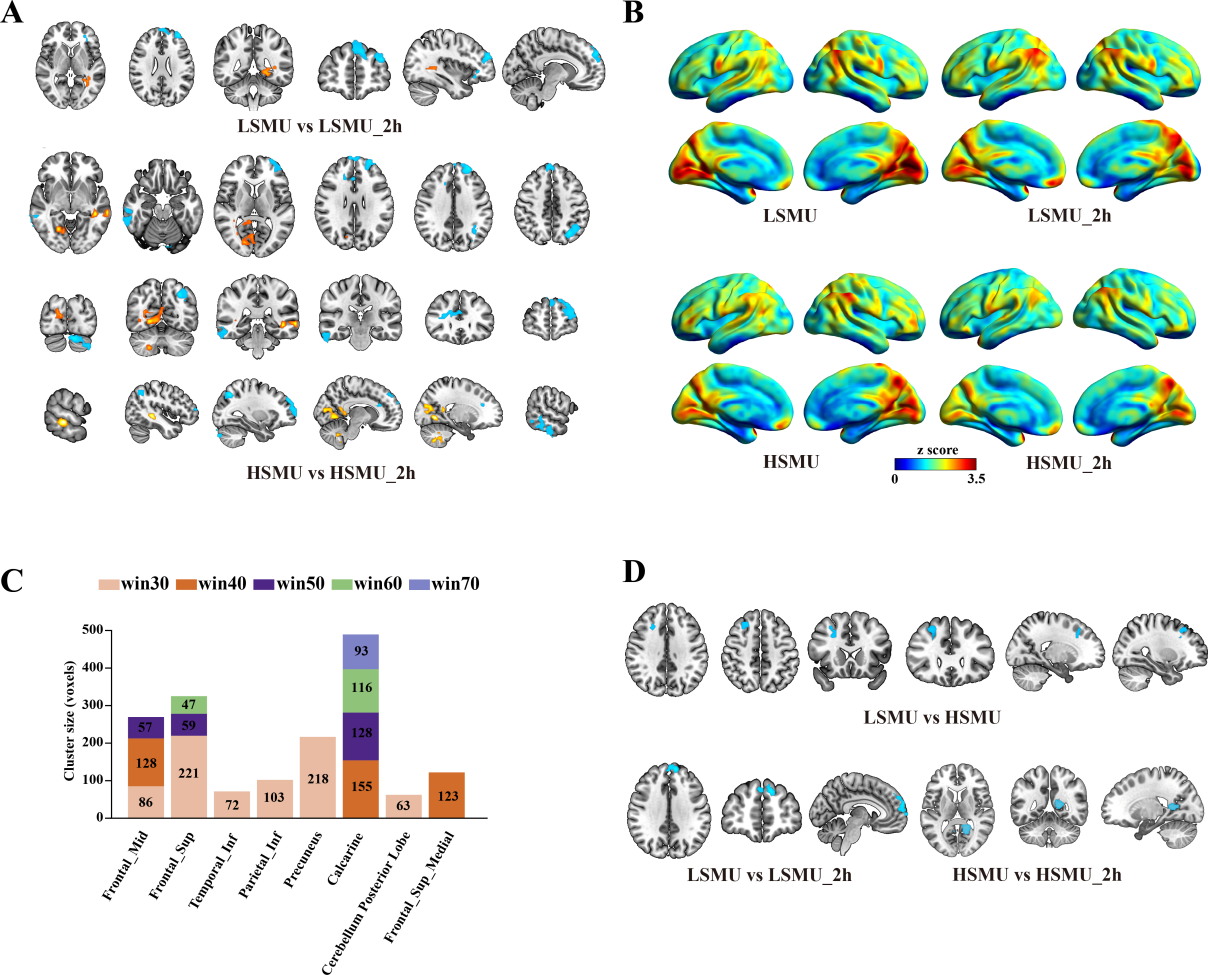
Group differences in static amplitude of the low-frequency fluctuations (sALFF) and dynamic amplitude of low-frequency fluctuations (dALFF) variability. (A) sALFF comparison results. Orange indicates increased sALFF in the latter relative to the former, and blue indicates decreased amplitude of low-frequency fluctuations (ALFF) values. (B) Spatial distribution of dALFF variability in the heavy social media users (HSMU) and the light social media users (LSMU) groups. (C) Validation results for different window sizes. (D) dALFF comparison results. Orange indicates increased dALFF in the latter relative to the former, and blue indicates decreased ALFF values. Frontal_Mid: middle frontal gyrus; Frontal_Sup: superior frontal gyrus; Frontal_Sup_Medial: medial superior frontal gyrus; HSMU_2h: heavy social media users group after the 2-hour social media use task; LSMU_2h: light social media users group after the 2-hour short social media use task; Parietal_Inf: inferior parietal lobule; Temporal_Inf: inferior temporal gyrus; TR: repetition time; win30: window length of 30 TRs; win40: window length of 40 TRs; win50: window length of 50 TRs; win60: window length of 60 TRs; win70: window length of 70 TRs.

There was no difference in the sALFF between the 2 groups. Compared with the LSMU group, the variance of dALFF in the right middle frontal gyrus was lower in the HSMU group (Figure 2D, voxel *P*<.005, cluster *P*<.05, and GRF corrected).

### Frontal Lobe Suppression After Short-Term SMU

After a 2-hour SMU task, the LSMU group showed increased sALFF in the temporal lobe but decreased sALFF in the left middle and superior frontal gyri. The HSMU group showed increased sALFF in the left middle temporal gyrus and right cerebellum 9 region but decreased sALFF in the right inferior temporal gyrus, left superior parietal gyrus, prefrontal cortex (PFC), and left cerebellar peduncle 2 area (Figure 2A, voxel *P*<.005, cluster *P*<.05, and GRF corrected). Figures 2B,C show the spatial distribution of dALFF variability and window-size validation. After a 2-hour SMU task, the LSMU group showed decreased variance of dALFF in the left superior medial frontal gyrus. In contrast, the HSMU group showed decreased variance of dALFF in the left middle frontal gyrus and increased variance in the right calcarine cortex (Figure 2D, voxel *P*<.005, cluster *P*<.05, and GRF corrected). Multimedia Appendix 1 showed results across different window sizes.

### Divergent FC Changes After Short-Term SMU

**Figure 3.**
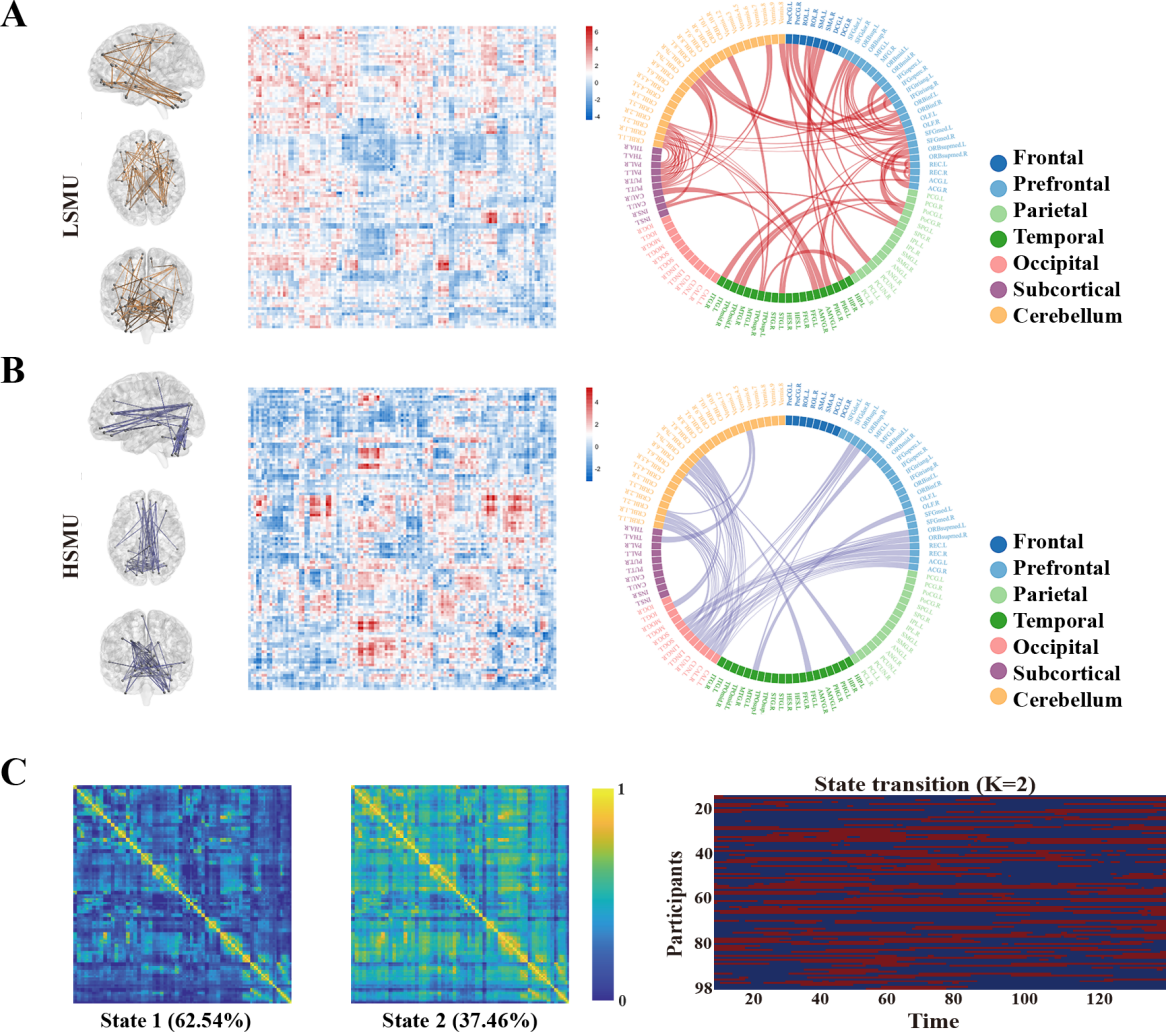
Group differences in static functional connectivity (sFC) and dynamic functional connectivity (dFC). (A) The light social media users (LSMU) group showed a significant increase in whole-brain sFC following short-term social media use (SMU), including nearly the entire resting-state subnetwork. (B) The heavy social media users (HSMU) group showed a significant decrease in whole-brain sFC after short-term SMU, mainly within the visual network (VN) and limbic system, between the VN and default mode network (DMN), and between the VN and cerebellum. All color labels in the legend (frontal, prefrontal, parietal, etc.) correspond directly to the anatomical regions of the nodes shown in the network diagrams. (C) Results of dFC with a window size of 40 TRs. The left graph shows the dFC cluster centroids using the Anatomical Automatic Labeling template, and the frequency of each state is marked on the figure. The right graph shows the transition matrix between 2 states. Horizontal coordinates indicate time points, and vertical coordinates indicate each participant’s 2 sessions combined.

After a 2-hour SMU task, LSMU showed increased sFC (excluding the VN; Figure 3A, *P*<.05, and NBS), while HSMU exhibited decreased sFC in prefrontal, occipital, temporal, and cerebellar regions, involving connections between the VN and the limbic system, DMN, and cerebellum (Figure 3B, *P*<.05, and NBS). No significant dFC differences emerged between groups. k-means clustering identified 2 states of dFC (Figure 3C, state 1 accounted for 62.54% of all windows, and state 2 accounted for 37.46%). State 1 exhibited loose connectivity except in visual or auditory regions (calcarine, cuneus, lingual gyrus, superior occipital gyrus, middle occipital gyrus, Heschl gyrus, and superior temporal gyrus), while state 2 showed tight connectivity across all networks except the cerebellum.

### Group Differences in EEG Microstates

**Figure 4.**
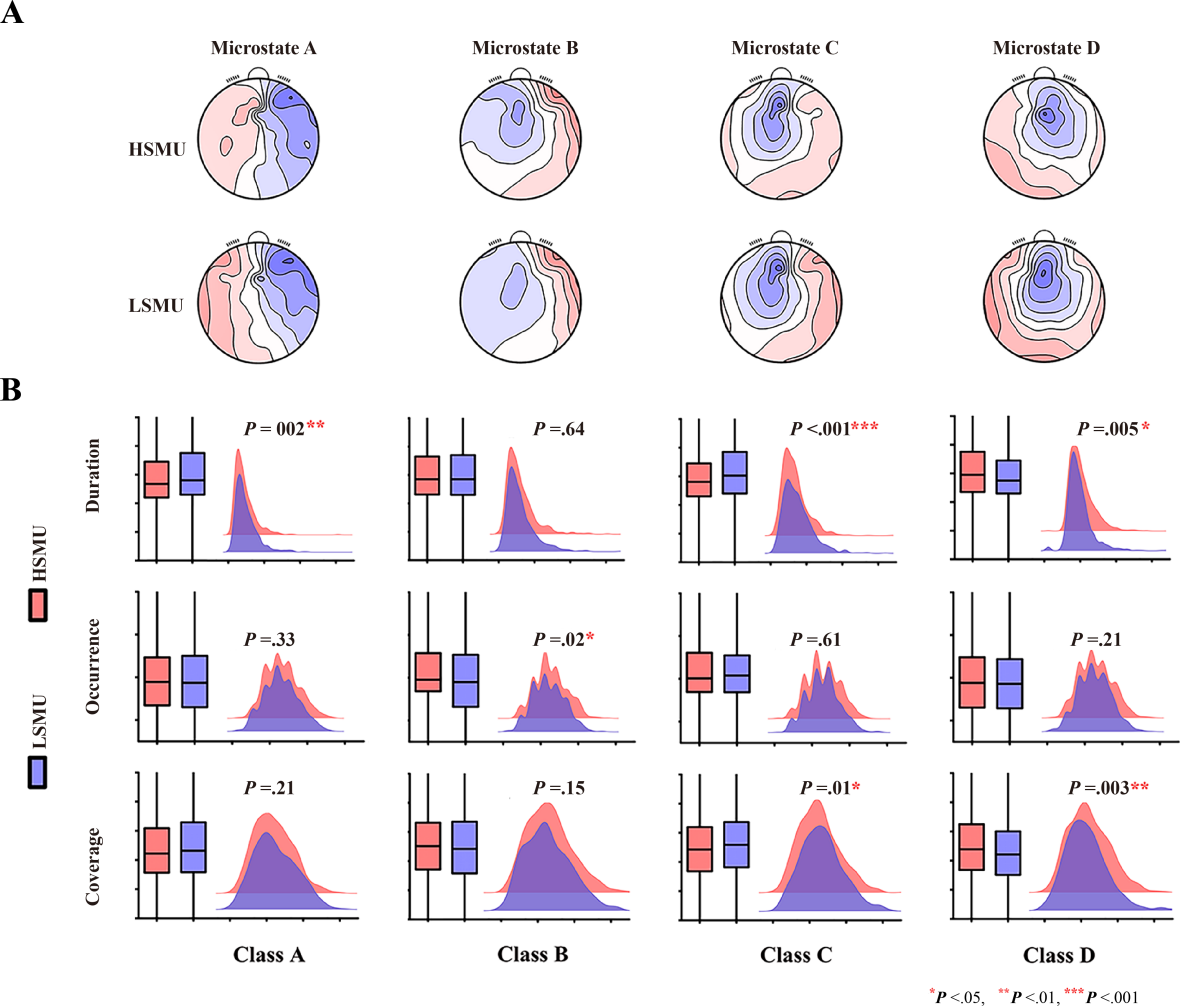
Microstate results. (A) The topographic map of the heavy social media users (HSMU) and light social media users (LSMU) groups. The color scale indicates opposite polarities, with red and blue representing the 2 ends of the potential distribution. The values are normalized by global field power (GFP) and expressed in arbitrary units. The polarity shown corresponds to the instantaneous potential at the GFP peak, and the inverted polarity of the same topographic map is considered to belong to the same microstate class. (B) Differences between the groups in the duration, occurrence, and coverage of the 4 microstates.

A total of 15 participants from the HSMU group and 15 participants from the LSMU group were deleted from the microstate analysis after the EEG preprocessing without introducing significant differences in demographic features or SMU. The topographic maps of the HSMU and LSMU groups are shown in Figure 4A. Microstate classes A to D were similar to the standard microstate maps reported earlier [38,39]. The 4 microstate classes accounted for 88.17% and 88.36% of the global explained variance in the HSMU and LSMU groups, respectively. The differences between groups with duration, occurrence, and coverage for the 4 microstate classes are shown in Table 2 and Figure 4B. After a 2-hour SMU task, the duration of microstate A in the HSMU group was shorter than that in the LSMU group. The duration was shorter and coverage of microstate C was also lower. Meanwhile, the HSMU group showed higher occurrence of microstate B, as well as longer duration and higher coverage of microstate D.

**Table 2.** Microstate temporal characteristics parameters and between-group comparisons (n=15)^a^.

Parameters	HSMU^b^, median (IQR)	LSMU^c^, median (IQR)	z value	*P* value
Duration				
Microstate A	53.4 (44.0‐69.3)	56.4 (46.1‐74.6)	−3.17	.002^d^
Microstate B	57.3 (46.4‐73.3)	57.0 (46.0‐74.0)	0.47	.64
Microstate C	56.0 (45.6‐69.3)	60.3 (48.0‐77.0)	−3.87	<.001^e^
Microstate D	58.6 (47.0‐74.5)	55.3 (46.0‐69.0)	2.79	.005^d^
Occurrence				
Microstate A	3.8 (2.7‐4.9)	3.7 (2.6‐5.0)	0.98	.33
Microstate B	3.9 (3.4‐5.2)	3.8 (2.6‐5.1)	2.26	.02^f^
Microstate C	4.0 (3.4‐5.2)	4.2 (3.4‐5.1)	−0.51	.61
Microstate D	3.8 (2.6‐5.0)	3.7 (2.6‐4.9)	1.25	.21
Coverage				
Microstate A	22.2 (15.6‐30.9)	23.2 (15.8‐33.0)	−1.24	.21
Microstate B	25.1 (17.1‐33.2)	24.1 (15.8‐33.5)	1.44	.15
Microstate C	24.3 (16.8‐32.0)	25.9 (18.2‐33.7)	−2.54	.01^f^
Microstate D	23.9 (16.8‐32.5)	22.1 (15.1‐30.0)	2.96	.003^d^

a Sample sizes reflect the number of participants whose electroencephalography data remained after artifact rejection.

b HSMU: heavy social media users.

c LSMU: light social media users.

d *P*<.01.

e *P*<.001.

f *P*<.05.

## Discussion

### Principal Findings

This is a cross-sectional study with acute experimental manipulation that includes fMRI and EEG to analyze resting-state functional brain patterns and brain dynamics characteristics in study participants with different SMU habits. Given the exploratory nature of our approach (voxel-level *P*<.005 and a 40 TR sliding window), the following findings should be interpreted as preliminary. (1) The HSMU group had lower frontal lobe activity than the LSMU group. (2) Participants with different SMU backgrounds showed differing responses to the same SMU task: the LSMU group showed decreased frontal lobe activity and increased temporal lobe activity. The HSMU group showed decreased activity in the frontal lobe, PFC, and parietal lobe, as well as altered activity in the temporal lobe and cerebellum. (3) SMU relies more on the VN, resulting in reduced connectivity with other network functions. (4) EEG microstate analysis showed alterations in microstates A, C, and D in the HSMU group.

The HSMU and LSMU groups exhibited distinct differences in baseline local brain activity, primarily localized in the frontal lobe. Intriguingly, the HSMU group demonstrated significantly reduced dALFF variability in this region, suggesting diminished dynamic flexibility within their attentional control network. Notably, the frontal lobe is known to encode social-emotional information in a contextually stable manner [40,41]. Chronic daily exposure to large volumes of social media content in the HSMU group may lead to rigid processing patterns in this region, thereby reducing temporal fluctuations in spontaneous neural activity. These baseline findings further imply that SMU is associated with a reduced brain capacity to integrate attentional control with social cognitive functions.

After a 2-hour SMU task, both groups exhibited an increase in sALFF within the temporal lobe. Although SMU is characterized by the processing of brief and discontinuous information, it still necessitates fundamental language decoding and preliminary semantic integration—processes that predominantly rely on temporal lobe activation [42]. More importantly, the LSMU group displayed reduced local brain activity, particularly in the frontal lobes, with decreased activity in the middle and superior frontal gyri. SMU is associated with inattention and mind wandering [43]. This may be attributed to the fragmented nature of social media content, as fragmented reading behavior has been negatively correlated with attentional capacity [44]. One study further characterized fragmented reading as a form of inattentive reading during which the mind tends to wander [45]. Given that the frontal lobe is a region critically involved in attentional control, such patterns of engagement may be reflected in reduced frontal lobe activity. In contrast, the HSMU group showed more extensive abnormalities, including sALFF reductions in the parietal lobe, PFC, and bidirectional cerebellar changes. The PFC, critical for cognitive control, task switching, and attention allocation [46-48], exhibited patterns similar to those observed in short video addiction [49], suggesting that acute SMU is associated with reduced emotional and attentional regulation. Additionally, cerebellar activity changes in the HSMU group align with its roles in time perception, working memory, social cognition, and emotional processing [50-53]. Collectively, prolonged SMU appears to deplete neural compensatory reserves, resulting in broader and more severe posttask abnormalities across cognitive and emotional networks. These disruptions may adversely affect information processing [54-56].

FC analysis revealed distinct patterns of neural network interactions between groups following SMU. While no baseline differences were observed between the HSMU and LSMU groups, the acute experimental manipulation analysis demonstrated divergent posttask connectivity patterns. The LSMU group exhibited widespread increases in resting-state subnetwork connectivity throughout the brain, with the notable exception of the VN. In contrast, the HSMU group showed significant decreases in sFC between the VN and several key networks, including the limbic system, DMN, and cerebellum. These findings support existing evidence that social media exposure leads to repetitive visual stimulation [45], potentially resulting in rigid information processing patterns that prioritize superficial VN engagement over deeper cognitive integration [57,58]. Such neural adaptation may impair the VN’s capacity for efficient cross-network communication.

Microstate analysis revealed distinct neural processing patterns between groups. Consistent with amplitude of low-frequency fluctuations findings highlighting temporal lobe involvement, microstate A showed significantly shorter duration in the HSMU group. Evidence from a previous study combining EEG microstates and fMRI suggests that microstate A is related to temporal lobe activity [59]. In the context of resting-state measurement immediately following the social media task, this finding may reflect a neural aftereffect of prior continuous visual information processing, manifesting as a tendency for rapid state transitions rather than sustained engagement of temporal lobe regions. The HSMU group also demonstrated shorter duration and lower coverage of microstate C, indicating suppressed DMN activity—a finding consistent with the observed DMN-VN FC reduction and potentially reflecting diminished self-referential processing capacity as an aftereffect of prior sustained external information demands during the task [60]. Conversely, HSMU participants exhibited longer duration and higher coverage of microstate D, which may represent compensatory neural exhaustion rather than enhanced dorsal attention network functionality. While these microstate D alterations bear similarity to patterns observed in attention deficit hyperactivity disorder [61] and Alzheimer disease [62], the underlying mechanisms in HSMU likely reflect adaptive rather than neurodegenerative processes. Given that EEG data were collected only after the 2-hour task, without a pretask baseline, the observed microstate differences may reflect a combination of preexisting group characteristics. These preliminary findings warrant further investigation to elucidate their functional significance.

The current findings align with neurocognitive models of the impact of SMU. Reduced frontal regional activity in HSMU reflects impaired executive control, as the frontal lobe mediates attention and cognitive regulation [63]. Posttask sALFF decreases in the parietal and prefrontal cortices, together with bidirectional changes in the cerebellum, further suggest compromised multisensory integration [64]. The enhanced network connectivity in LSMU may indicate adaptive cognitive resource allocation, whereas reduced visual-DMN sFC in HSMU implies disrupted internal-external attention modulation [65]. Decreased duration and coverage of microstates A and C align with findings linking these microstates to DMN function and cognitive processing [66], supporting neurofunctional adaptations to high social media engagement. Beyond these findings, this study offers 3 technical contributions. First, we integrated sFC, dFC, and EEG microstates into a synergistic framework. sFC captures stable interactions but overlooks temporal variability; dFC tracks transient fluctuations; EEG microstates provide millisecond-scale dynamics [67,68]. By correlating these techniques, we characterized brain function across temporal scales. Second, we implemented network-labeled microstate interpretation linking microstates to specific functional networks, going beyond traditional unsupervised clustering [69]. Third, we pioneered this framework to investigate SMU-related neuroplasticity by combining dFC and microstates—techniques highly sensitive to rapid cognitive and attentional shifts—uncovering novel markers (altered microstate A and C duration) obscured by conventional static analyses [70,71].

### Future Work

The distinct neural patterns observed between HSMU and LSMU suggest that future research could explore the potential reversibility of these alterations through longitudinal tracking or intervention studies. Noninvasive brain stimulation techniques, such as TMS or tES, are promising tools for this purpose. The reduced frontal lobe activity and disrupted VN connectivity identified in HSMU users provide potential spatial targets for neuromodulation. The altered EEG microstates, particularly A and C, may further serve as electrophysiological biomarkers for monitoring treatment response in such interventions. Additionally, investigating whether these neural changes extend to other forms of digital media use would also help clarify the specificity of the observed effects. Additionally, future studies employing EEG-fMRI data fusion could further elucidate the relationship between spatial and temporal neural dynamics in the context of SMU. It should also be noted that our exploratory choice of the 40-TR sliding window used in our dynamic analyses primarily captures slow low-frequency fluctuations. Faster cognitive shifts, such as mind-wandering or attentional lapses, may operate on shorter timescales and could be overlooked with this approach. Future studies employing shorter sliding windows (eg, 15‐30 TRs) or event-related designs would help characterize these rapid neural dynamics.

### Limitations

The present study has some limitations. First, as a cross-sectional study with an acute experimental manipulation study, we used a voxel-level threshold of *P*<.005 (GRF-corrected) for fMRI analysis, and the EEG microstate comparisons between groups were not corrected for multiple comparisons. These correction strategies are relatively lenient. Thus, our imaging findings are exploratory. Future studies with larger samples and more rigorous statistical thresholds are needed to confirm our findings. Second, we chose 40 TRs for the main analysis and presented additional window lengths in Multimedia Appendix 1. Given the lack of consensus on the optimal window size, future studies may further examine this parameter. Third, the study did not include a non–social media control condition, which limits our ability to fully differentiate the effects of SMU from general cognitive fatigue. Future research incorporating a control task would help clarify the specificity of the observed neural changes. Fourth, our study was based on analyses of resting-state neuroimaging, which may not fully express SMU in the dynamic evolution of the cognitive processing model. Future studies should capture brain activity during SMU and consider combining multimodal neuroimaging with behavioral measurements. Fifth, only male participants were included in the current study due to a significant sex imbalance in the original cohort. Future studies with balanced sex representation are needed to improve the generalizability of the findings. Sixth, a relatively high number of participants were excluded from the EEG microstate analysis due to artifacts. Future studies should focus on improving data acquisition quality to minimize data loss. Seventh, our experimental condition required participants to log out and view random hot topics rather than personalized algorithmic content, which reduces ecological validity. As a result, the task may be more akin to a continuous reading or visual-stimulation task than to naturalistic SMU.

### Conclusions

This study employed a multistage concurrent EEG-fMRI design to investigate neural alterations associated with different patterns of SMU. The findings revealed that HSMU groups exhibited distinct neural characteristics, including reduced dynamic brain activity in the frontal lobe, disrupted connectivity of the VN, and altered temporal dynamics in EEG microstates. These findings provide a multimodal characterization of the neural correlates of SMU.

## Supplementary material

10.2196/86963 Multimedia Appendix 1 Information on participant recruitment and grouping; data acquisition and preprocessing; group comparisons of dynamic amplitude of low-frequency fluctuations variability at window sizes of 30, 40, 50, 60, and 70 TRs; the experimental interface and setting for the 2‑hour Weibo task; and dynamic functional connectivity clustering states identified by window sizes of 30, 50, 60, and 70 TRs, respectively.

## Abbreviations

dALFF: dynamic amplitude of low-frequency fluctuations
dFC: dynamic functional connectivity
DMN: default mode network
EEG: electroencephalography
EEGLAB: electroencephalography laboratory
FASTR: functional magnetic resonance imaging artifact slice template removal
FC: functional connectivity
fMRI: functional magnetic resonance imaging
FMRIB: functional magnetic resonance imaging of the brain
GRF: Gaussian random field
HSMU: heavy social media users
LSMU: light social media users
MNI: Montreal Neurological Institute
MR: magnetic resonance
NBS: network-based statistics
PFC: prefrontal cortex
sALFF: static amplitude of low-frequency fluctuations
sFC: static functional connectivity
SMU: social media use
tES: transcranial electrical stimulation
TMS: transcranial magnetic stimulation
TR: repetition time
VN: visual network
