# Supplementary material for: Effects of Social Media Use on Brain Dynamics in Young Male Adults: Multistage Concurrent Electroencephalography–Functional Magnetic Resonance Imaging Study
Source: JMIR Med Inform. 2026 Aug 6;14:e86963. doi: 10.2196/86963 (PMC13446798; doi:10.2196/86963)
Supplement: Supplementary file 1 [file medinform-v14-e86963-s001.docx]

**Supplementary materials**

**Participant’s recruitment and grouping**

We aimed to recruit two groups of participants: heavy and light social media users. Given that there is currently no definitive standard for distinguishing between heavy social media use (HSMU) and light social media use (LSMU), we ranked the cohort by daily time spent on social media. We aimed to recruit a similar number of participants, with approximately 40 participants per group, from both ends, with the top as HSMU and the bottom as LSMU. A natural break was observed between the 35th subject (1.8 h) and the 36th subject (1 h). Therefore, we recruited the top 35 subjects for the HSMU group to improve consistency. For the LSMU group, the daily time spent on social media of the bottom 40th subject (0.7 h) was the same as the bottom 41st and 42nd subjects. Thus, we recruited the bottom 42 subjects to ensure consistency. Finally, two groups were recruited: the top 35 subjects with the heaviest daily social media use as the HSMU group, and 21 subjects randomly selected from the bottom 42 subjects as the LSMU group. The data from the other bottom 21 subjects were not used, as they are not the focus of the current research. After data preprocessing, five HSMU participants were excluded. Four were excluded due to excessive head motion, and one due to claustrophobia. The final HSMU sample was 30. In the LSMU group, one participant was excluded due to head motion and one withdrew, leaving a final LSMU sample of 19.

**Data acquisition and preprocessing**

High-resolution T1-weighted images were acquired using a three-dimensional brain volume (3D-BRAVO) sequence with the following parameters: echo time (TE) = 3.2 ms, repetition time (TR) = 8.2 ms, flip angle (FA) = 12°, field of view (FOV) = 256 × 256 mm², matrix = 256 × 256, slice thickness = 1.0 mm, and 188 slices. T1-weighted imaging data were preprocessed using the Computational Anatomy Toolbox (CAT12) within the Statistical Parametric Mapping (SPM12) framework implemented in MATLAB. Spatial normalization to Montreal Neurological Institute (MNI) space was performed using the DARTEL algorithm. Brain tissue extraction was conducted using the Brain Extraction Tool (BET). The normalized T1 data were subsequently used to register resting-state blood oxygenation level-dependent (BOLD) data to standard space.

**Group differences in dALFF variability**

**Table S1**

Group differences in dALFF variability with different window sizes.

| **Group** | **Window size** | **Cluster size (voxels)** | **MNI coordinate** | **t-value** | **Region** |
| --- | --- | --- | --- | --- | --- |
| **LSMU vs HSMU** |  |  |  |  |  |
| LSMU > HSMU | 30 TRs | 63 | -48, -66, -51 | 4.81 | Cerebellum Posterior Lobe |
|  |  | 67 | 24, 30, 48 | 3.98 | Frontal_Sup_R |
|  | 40 TRs | 61 | 24, 24, 36 | 4.26 | Frontal_Mid_R |
|  | 50 TRs | 57 | 24, 24, 36 | 4.29 | Frontal_Mid_R |
|  | 60 TRs | - | - | - | - |
|  | 70 TRs | - | - | - | - |
| **LSMU vs LSMU_2h** |  |  |  |  |  |
| LSMU > LSMU_2h | 30 TRs | 154 | -12, 54, -33 | 6.25 | Frontal_Sup _L |
|  | 40 TRs | 123 | -3, 66, 24 | 5.48 | Frontal_Sup_Medial_L |
|  | 50 TRs | 59 | -12, 54, 33 | 5.23 | Frontal_Sup _L |
|  | 60 TRs | 47 | -12, 54, 33 | 4.66 | Frontal_Sup _L |
|  | 70 TRs | - | - | - | - |
| **HSMU vs HSMU_2h** |  |  |  |  |  |
| HSMU > HSMU_2h | 30 TRs | 72 | 57, -36, -15 | 4.73 | Temporal_Inf_R |
|  |  | 86 | -33, 48, 33 | 4.37 | Frontal_Mid_L |
|  |  | 103 | -48, -60, 42 | 4.73 | Parietal_Inf_L |
|  | 40 TRs | 67 | -33, 48, 33 | 4.38 | Frontal_Mid_L |
|  | 50 TRs | - | - | - | - |
|  | 60 TRs | - | - | - | - |
|  | 70 TRs | - | - | - | - |
| HSMU < HSMU_2h | 30 TRs | 218 | 15, -48,12 | 4.77 | Precuneus_R |
|  | 40 TRs | 155 | 18, -48, 9 | 4.93 | Calcarine_R |
|  | 50 TRs | 128 | 18, -48, 9 | 4.67 | Calcarine_R |
|  | 60 TRs | 116 | 18, -54, 6 | 4.46 | Calcarine_R |
|  | 70 TRs | 93 | 18, -54, 6 | 4.54 | Calcarine_R |

Abbreviations: MNI, Montreal Neurological Institute; LSMU, light social media use; HSMU, heavy social media use; LSMU_2h, light social media use group after a 2-hour social media use task; HSMU_2h, heavy social media use group after a 2-hour social media use task.

**Figure S1** Participants could only browse the "hot issues" on Weibo for 2 hours (HSMU group average daily SMU time) while logged out. This module randomly displayed the latest hot topics rather than those based on personal preferences, avoiding deviations caused by different programs. No other applications were allowed during the task, which was performed in a quiet laboratory room to prevent potential bias.

**Figure S2.** Results of dFC when the window size was 30TRs.

**Figure S3.** Results of dFC when the window size was 50TRs

**Figure S4.** Results of dFC when the window size was 60TRs.

**Figure S5.** Results of dFC with a window size of 70 TRs.
